# A Few Cases Illustrative of the Ill Effects of Onanism

**Published:** 1846-07

**Authors:** Edward L. Baker

**Affiliations:** Fort King, Florida


*A few cases illustrative of the ill effects of Onanism.* By EDWARD L. BAKER, M. D., of Fort King, Florida.—Onanism, or the sin of Onan, is from a perverted passage in the Pentateuch. The discharge of semen from a preternatural stimulus is the vice, it is said, of the solitary monk, and perhaps, of other recluses, to whom more natural enjoyments are denied. It is a habit of the most destructive tendency, enervating, in the highest degree, both the body and mind, as the following cases will illustrate. Nature seems to have fixed a strong mark on those disposed to every unnatural enjoyment, however secret their practices may be, and so indelible is this mark, that they cannot escape detection from that *tact* which has been peculiarly distinguished by the term of *sensus medicus.* In general, the countenance is sallow, with a peculiar dejection in the look. The voice is hurried and unsteady; the face sometimes covered with dark coloured blotches or flushed, particularly when looked upon full in the face, and the whole frame displays a peculiar debility. The dejection, at times, almost amounts to insanity, and every effort appears to threaten instant death. The tremor and apprehension prevent the natural enjoyments, by which they might be otherwise weaned from this destructive habit; and the whole life is alternated with doubts, apprehensions and despair. Unfortunately, the practice is seldom forsaken—at least, we have reason to think so.

The apprehensions of discovery and the despair, render these unfortunate persons the dupes of quacks. Regular practice exhausts the whole class of tonics and stimulants with little effect. The warm balsams, of which the quack medicines consist, are either rejected from the hands of the physician, or not continued a sufficient time; and even our best remedies, do not fix the imagination so strongly as the Solar tincture or the balm of Gilead. It is probable, if not too long continued, a prudent marriage might recover the patient; but it would be unjust, cruel, and impolitic, to condemn a healthy young woman to the shadow of a man. I will now give the report of a few cases.

Case 1st. A man aged about 30, was brought from a distance and placed under my care. I found him in the following situation: A very large and ample frame, his muscles soft and flabby, and very much emaciated; he had no appetite, almost loathed food of all kinds, had no energy either of mind or body, but would sit in one position nearly all day; he appeared even reluctant to answer in monosyllables, felt convinced that he could not live, was troubled at times with great thirst for cold drinks; his cheeks were very much flushed, could not look in the face even his most intimate friends, but always inclined his eyes downwards, complained at times, particularly after eating, (and that too of the most simple articles of food,) of pyrosis. Pulse frequent, but weak; almost constant cephalalgia, with tenderness of epigastrium under pressure.

I was led to believe his a case of genuine dyspepsia, or chronic inflammation of the stomach, and treated it accordingly. Sometime, however, after this, I accidentally discovered from a friend of his, that he had been addicted to the most disgusting practice of secret pollution from his boyhood. I then adopted a moral course of treatment, endeavoured to reason the case with him, informed him that this was the cause of his disease, that it was this that was killing him, and that the only chance for his life, was for him to leave off this most diabolical habit. I even went so far as to pustulate the penis with the ung. tart. antim., but this did not prevent him, and he died in about ten days after.

Case 2d. A physician of considerable celebrity had a negro man placed under his care: he kept him in his yard, that he might watch more closely the effects of his medicine. The patient was very much emaciated, constant cephalalgia, palpitation of the heart, pain and some swelling, with extreme weakness in the joints of the inferior extremities—he was compelled to use crutches—and, as in the former case, was very much dejected, and was firmly of opinion that he would die; he was treated by his physician for disease of the heart, and also for spinal irritation. After some time, finding he made no progress in relieving his disease, he requested me to see him. I at once, and without hesitation, (after examination and observing his countenance) stated to my friend that I suspected him of being addicted to onanism. He accordingly took care to watch him, and detected him in the act. He at once told him he would be compelled to castrate him unless he would quit this practice. He also applied the ointment as used in the preceding case, and gave tonics, stimulants, cold bath, &c. This patient ultimately recovered, and is now a healthy and valuable servant. He has since acknowledged to his physician that he had been addicted to the practice for seven or eight years, and that he not unfrequently operated in this manner eight or nine times in the course of the twenty-four hours. Both of the above patients complained of pain in the occiput and were very nervous.

Case 3d. The following case was related to me by a physician who had been intimate with the young man. This friend, he says, had received a good education from a father who was very wealthy and stood high in society. The young man, however, became addicted to this vice, was gloomy and taciturn, shunned his friends, and indeed all society, sought solitude, and his eyes were always cast down—in fact he was to all appearance miserable. His father one day mentioned to him that, at such a time he would give him a certain amount of money, for the purpose of commencing business for himself, and that he would render him all the assistance in his power. The idea of being compelled to mingle in society (as he would have been forced to do if he went into business,) prayed so much upon his mind, that before the day arrived for him to receive his gift, he committed suicide.

I am acquainted with three idiots, and in each of the cases, it is a fact known to all who are acquainted with their histories, that they are daily addicted to this vice.

I should be happy to hear from others on this important subject—I feel convinced that the great majority of the cases of chronic diseases which we meet with in practice, can be traced to this cause. There has been so little written on this subject in this country, that physicians rarely suspect a case of this kind unless they are made acquainted with it by some accidental occurrence.—*Ibid.*

